# Increased Endothelial Progenitor Cell Levels are Associated with Good Outcome in Intracerebral Hemorrhage

**DOI:** 10.1038/srep28724

**Published:** 2016-06-27

**Authors:** Juan Pías-Peleteiro, María Pérez-Mato, Esteban López-Arias, Manuel Rodríguez-Yáñez, Miguel Blanco, Francisco Campos, José Castillo, Tomás Sobrino

**Affiliations:** 1 Clinical Neurosciences Research Laboratory, Department of Neurology, Hospital Clínico Universitario, Health Research Institute of Santiago de Compostela (IDIS), University of Santiago de Compostela, Santiago de Compostela, Spain

## Abstract

Circulating endothelial progenitor cells (EPCs) play a role in the regeneration of damaged brain tissue. However, the relationship between circulating EPC levels and functional recovery in intracerebral hemorrhage (ICH) has not yet been tested. Therefore, our aim was to study the influence of circulating EPCs on the outcome of ICH. Forty-six patients with primary ICH (males, 71.7%; age, 72.7 ± 10.8 years) were prospectively included in the study within 12 hours of symptom onset. The main outcome variable was good functional outcome at 12 months (modified Rankin scale ≤2), considering residual volume at 6 months as a secondary variable. Circulating EPC (CD34^+^/CD133^+^/KDR^+^) levels were measured by flow cytometry from blood samples obtained at admission, 72 hours and day 7. Our results indicate that patients with good outcome show higher EPC numbers at 72 hours and day 7 (all p < 0.001). However, only EPC levels at day 7 were independently associated with good functional outcome at 12 months (OR, 1.15; CI95%, 1.01–1.35) after adjustment by age, baseline stroke severity and ICH volume. Moreover, EPC levels at day 7 were negatively correlated to residual volume (r = −0.525; p = 0.005). In conclusion, these findings suggest that EPCs may play a role in the functional recovery of ICH patients.

Intracerebral hemorrhage (ICH) represents 10–15% of all strokes^1^. Early mortality ranges between 32% and 52% within the first 30 days, only 10% of patients will live independently after 1 month, and only one in five patients will be autonomous at 6 months^2^. Despite being the most severe cerebral vascular disorder, there is no specific pharmacological treatment. Early surgery, even if does not increase the disability or death rate at 6 months, might have only a small clinically relevant survival advantage for patients with spontaneous superficial ICH without intraventricular hemorrhage^3^. Therefore, it is imperative to search for new therapeutic options. In this regard, the beneficial effects of bone marrow-derived progenitor cells (BMPCs) have been demonstrated in animal models of ICH, as evidenced by reduced tissue loss, immature neuron formation, synaptogenesis, neuronal migration, and neurological improvement^4^,^5^,^6^,^7^. In clinical studies regarding patients after acute ischemic stroke, the increase of circulating endothelial progenitor cells (EPCs), a subtype of BMPCs, has been associated with good neurological and functional outcome, reduced infarct growth and neurological improvement^8^,^9^,^10^,^11^,^12^,^13^. Likewise, high blood levels of circulating CD34^+^ progenitor cells have been associated with improved functional outcome at three months and reduced brain injury in patients with primary ICH^14^. Furtheremore, a recent study involving 16 patients with ICH has shown increased levels of circulating EPCs^15^. However, the role of circulating EPCs in the recovery of ICH patients is largely unknown. Our aim was therefore to study the influence of circulating EPCs in the outcome of ICH.

## Results

We prospectively studied 46 patients with primary ICH (71.7% male, mean age 72.7 ± 10.8 years). Median [quartiles] NIHSS score at admission was 11 [5,16], and mean time from stroke onset was 4.7 ± 4.6 hours.

### Primary Outcome

**Figure 1:**
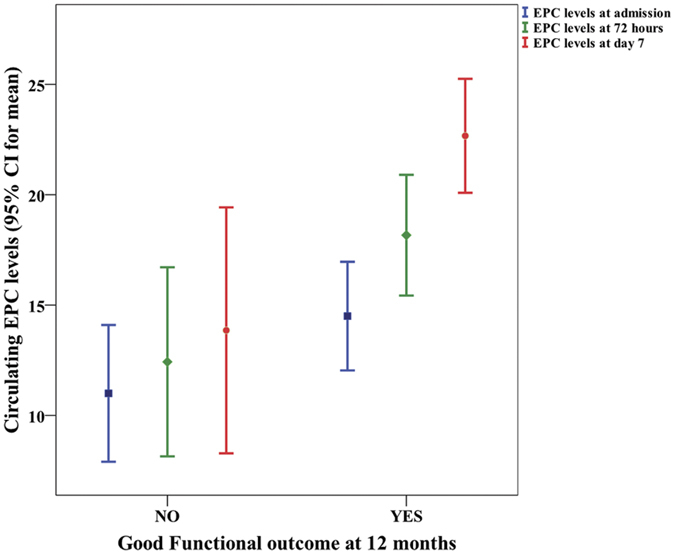
Temporal profile of mean circulating EPC numbers in ICH patients with good or poor outcome at 12 months.

Table 1 shows the main characteristics of patients classified by outcome groups. Nineteen (41.3%) patients showed good functional outcome at 12 months. Patients with good functional outcome were younger and showed milder stroke severity, lower rate of ventricular extension and leukoaraiosis, and smaller ICH and edema volumes at admission. Likewise, patients with good outcome (n = 19) showed higher EPC numbers at 72 hours (18.2 ± 5.5 vs. 11.2 ± 7.3; p = 0.003) and at day 7 (22.7 ± 5.3 vs. 13.5 ± 9.1; p = 0.001), but not at admission (14.2 ± 5.1 vs. 10.9 ± 6.1; p = 0.108) (Fig. 1). However, only EPC levels at day 7 were independently associated with good functional outcome at 12 months (OR, 1.15; CI95%, 1.01 to 1.35, p = 0.039) after adjustment by age, baseline stroke severity, and ICH volume (Table 2).

### Secondary Outcomes

**Figure 2:**
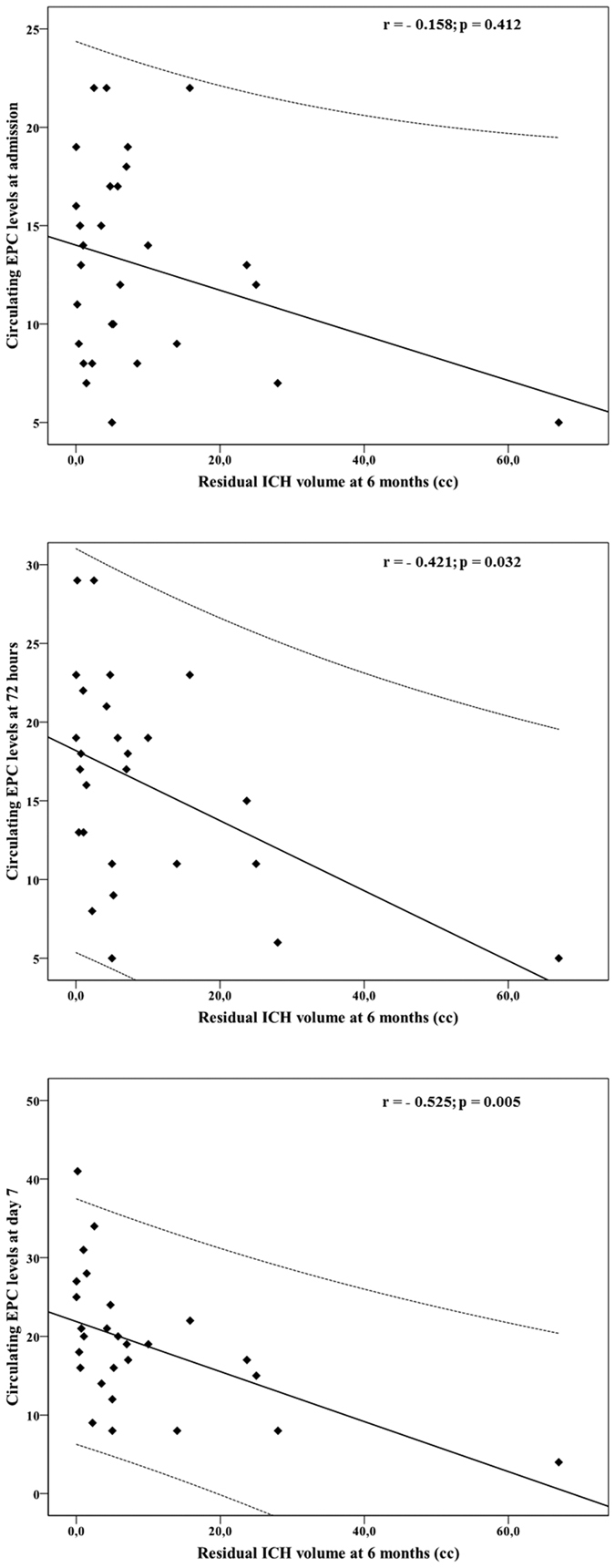
Scatterplot showing the correlation of circulating EPCs at admission, 72 hours and day 7 with ICH residual volume at 6 months. No correlation was found between circulating EPC levels at admission and ICH residual volume at 6 months (Pearson correlation coefficient, r = −0.158; p = 0.412). However, an exponential relation was found between ICH residual volume and EPC levels at 72 hours (r = −0.421; p = 0.032), and at day 7 (r = −0.525; p = 0.005).

Figure 2 shows the correlation of circulating EPCs at admission, 72 hours and day 7 with ICH residual volume at 6 months. No correlation was found between circulating EPC levels at admission and residual ICH volume at 6 months (Pearson correlation coefficient, r = −0.158; p = 0.412). However, an exponential negative correlation was found between residual ICH volume and EPC levels at 72 hours (r = −0.421; p = 0.032), and at day 7 (r = −0.525; p = 0.005). In the multivariate analysis, both circulating levels of EPCs at 72 hours (B, −1.01; CI95%, −1.79 to −0.21; p = 0.015) and especially at day 7 (B, −0.86; CI95%, −0.1.44 to −0.28; p = 0.005) were independently associated with residual ICH volume at 6 months. However, only circulating EPC levels at day 7 (B, −0.54; CI95%, −0.93 to −0.15; p = 0.009) were independenly associated with residual ICH volume at 6 months after adjusting by baseline stroke severity, as well as ICH and edema volumes at admission.

On the other hand, no correlation was found between circulating EPC levels at day 7 and NIHSS at admission (r = −0.237; p = 0.178). However, a significant negative correlation was observed between circulating EPC levels at day 7 and NIHSS at discharge (r = −0.607; p < 0.0001), at 3 months (r = −0.570; p < 0.0001) and at 12 months (r = −0.591; p < 0.0001).

## Discussion

### Main results and clinical relevance

To the best of our knowledge, this study is the first prospective analysis that evaluates the relationship between circulating levels of EPCs (defined as CD34^+^/CD133^+^/KDR^+^ cells) and brain injury in patients with ICH. Remarkably, circulating EPC levels at day 7 were independently associated with good functional outcome at 12 months. This favourable effect on the primary endpoint was supported by residual ICH volume reduction at 6 months and milder neurological deficits. Overall, these findings support cellular therapy with EPCs as a new therapeutic approach for patients with ICH, a severe vascular disorder with currently no specific pharmacological treatment.

### Possible mechanisms underlying EPC effects on recovery and ICH residual volume

We have demonstrated that circulating EPCS increase at day 7 in response to ICH, and that the greater the magnitude of this increases the better the clinical outcome at 12 months. These findings are in line with experimental studies demonstrating the beneficial effects of BMPCs in animal ICH models as evidenced by reduced tissue loss, immature neuron formation, synaptogenesis, neuronal migration and neurological improvement^4^,^5^,^6^,^7^. The fact that patients with good outcome showed higher EPC levels at day 7, but not at admission, supports the hypothesis that EPCs can mediate processes of chronic vessel repair and neurorepair. Furthermore, supporting this hypothesis, we found a negative correlation between increased levels of EPCs and smaller ICH residual volume at 6 months as well as milder neurological deficits. However, the mechanisms by which EPCs are associated with better functional outcome and with smaller residual ICH volume need further investigation. We hypothesize that this may occur through a triple mechanism: (a) the repairing of the damaged vessels through re-endotelization as previously shown in ventricular assist devices in humans^16^,^17^ (b) the developing of new vessels through neovascularization^18^,^19^,^20^ and (c) the paracrine action of EPCs promoting angiogenesis^21^,^22^,^23^,^24^. A possible fourth mechanism may add up in the acute phase of ICH, where EPCs may play an early role in protecting the blood-brain barrier (BBB), as some studies have indeed suggested in ischemic stroke^25^. This is consistent with other data from this study showing that reduced flow mediated dilation (which is positively correlated with EPCs; r = 0.602; p < 0.0001) holds a negative association with ICH and edema growth at 48–72 hours (data not shown).

On the other hand, previous studies by our group have observed that higher levels of growth factors at 72 hours after ICH are independently associated with a good functional outcome at 3 months, lesion volume reduction and relative neurological improvement ≥50% at day 90^26^. Likewise, we have previously demonstrated that higher levels of growth factors are associated with higher levels of circulating EPCs in human ischemic stroke^27^. Therefore, it is tempting to postulate that the beneficial effects of growth factors in ICH may be mediated by EPCs.

### Study limitations

This study has some limitations. First, we did not include a control group. Nevertheless, a previous study had already shown increased levels of circulating EPCs in ICH patients compared with a control group^15^. Another limitation is that we did not determine EPC levels at longer intervals after the acute phase of ICH, so we cannot conclude if good recovery was associated with an acute and transient increase in EPC levels or if it was the result of a sustained increase of these progenitor cells during the follow-up. However, the favourable effect of the EPC level increase at day 7 was independent of other important prognostic variables at baseline. Lack of consensus over optimal definition for EPCs and the best measurement method may justify many contradictory results concerning EPCs up to date. We have used CD34, CD133 and KDR markers, as well as flow cytometry analysis because both techniques are amply used and recommended for EPC characterization and counting^28^. We have not directly measured functionality of EPCs. Nevertheless, the most accepted method for EPC characterization is flow cytometry, and several studies in ischemic stroke patients have virtually come to the same results using this technique and cell culture for CFU-EC counts (which best expresses functionality of EPCs) suggesting a parallelism relationship between EPC numbers and functionality^8^,^10^,^11^.

## Conclusions. 

In conclusion, a higher increase in circulating EPC levels at day 7 is associated with a better outcome and reduced ICH residual volume in ICH patients. However, whether if EPCs are able to incorporate into brain hemorrhagic areas and promote neurorepair in humans remains to be clarified. Finally, the role of EPCs as a therapeutic tool able to promote chronic neurorepair of brain tissue damaged by an ICH needs to be further explored.

## Methods

### Study population and patient characteristics

Between October 2012 and February 2014, 59 consecutive patients with a first-ever primary non-traumatic ICH of less than 12 hours from clinical onset and previously independent for their daily living activities were prospectively included in the study. Patients with previously altered functional capacity (modified Rankin Scale (mRS) ≥ 1)^29^ (n = 3), chronic inflammatory diseases (n = 2), severe hepatic (n = 1) or renal (n = 1) diseases, cancer (n = 2) or infectious disease in the 15 days prior to inclusion (n = 1) were excluded. Furthermore, 2 patients did not accept their participation in the study, so a total of 46 patients were finally included.

This research was carried out in accordance with the Declaration of Helsinki of the World Medical Association (2008) and approved by the Ethics Committee of the Servizo Galego de Saúde. Informed consent was obtained from each patient or their relatives after full explanation of the procedures.

#### Clinical variables

All patients were admitted to an acute stroke unit and treated according to the guidelines of the Cerebrovascular Diseases Study Group of the Spanish Society of Neurology^30^. Etiological diagnosis was made according the Guidelines for the management of spontaneous intracerebral hemorrhage in adults from the American Heart Association/American Stroke Association Stroke Council, High Blood Pressure Research Council, and the Quality of Care and Outcomes in Research Interdisciplinary Working Group^31^. Medical history recording potential vascular risk factors, blood and coagulation tests, 12-lead ECG, and chest radiography was obtained at admission.

To evaluate neurologic deficit, the National Institute of Health Stroke Scale (NIHSS)^32^ was performed at admission, 24 and 72 hours, at discharge, and at 3 and 12 months. Functional outcome was evaluated at discharge and at 3 and 12 months by using the mRS^29^. Both NIHSS and mRS were evaluated by internationally certified neurologists.

Antihypertensive treatment with intravenous labetalol or urapidil was administered in case of systolic blood pressure >185 mmHg or diastolic blood pressure >105 mmHg. Low-dose subcutaneous heparin was used for the prevention of deep vein thrombosis and pulmonary thromboembolism.

#### Neuroimaging studies

A CT study was performed at admission, at 72 hours, between days 4^th^–7^th^, and at 6 months. ICH and perihematomal edema volumes were calculated by a standard planimetric method using a semiautomated process. The perimeter of appropriate high and low-attenuation zones was traced, calculating lesion areas for each slice which were multiplied by slice thickness to yield lesion volumes. The residual cavity volume of ICH at 6 months was determined using the same volumetric method described; each volume calculation was done three times, the mean value being taken as definitive.

ICH topography was classified as lobar when it predominantly affected the cortical or subcortical white matter of the cerebral lobes, or as deep when it was limited to the internal capsule, the basal ganglia or the thalamus. The presence of intraventricular extension of the hematoma was also recorded.

All neuroimaging evaluations were made by the same neuroradiologist, who had no knowledge of the patients’ clinical or laboratory results.

#### EPC quantification in peripheral blood by Flow Cytometry

Circulating EPC leves were measured by flow cytometry according to methods described elsewhere^33^ in blood samples obtained at admission, 72 hours and at day 7. Blood samples were processed within 1–2 hours after collection by a single researcher who had no knowledge of the patients’ clinical or radiological results. In brief, circulating EPCs were analyzed for the expression of specific surface antigens using direct flow cytometry (BD FACSAria IIu, BD, Franklin Lakes, NJ, USA). Cells were labelled with FITC-conjugated anti-CD34 (BD, Franklin Lakes, NJ, USA), PE-conjugated anti-KDR (R&D Systems, Minneapolis, MN, USA) and APC-conjugated anti-CD133 (clone AC133 from Miltenyi Biotec, Bergisch Gladbach, Germany) monoclonal antibodies. We considered EPCs as triple CD133^+^/CD34^+^/KDR^+^ staining cells in the mononuclear cell fraction. In all analyses, 5 × 10^5^ events were acquired, scored using a FACSAria IIu analyzer (BD, Franklin Lakes, NJ, USA), and processed using the PC FACSDiva software program (BD, Franklin Lakes, NJ, USA). Cell count was always expressed per 10^6^ events.

#### Outcome Variables

The primary endpoint was good functional outcome (mRS ≤ 2) at 12 months. ICH Residual volume at 6 months and the evolution of NIHSS score during the first 12 months were evaluated as secondary outcome variables.

#### Statistical Analysis

The statistical analysis was conducted in SPSS 18.0 (SPSS, Chicago, IL, USA) for Mac.

### Sample size calculation

Sample size was calculated using the statistical EPIDAT software (http://www.sergas.es/MostrarContidos_N3_T01.aspx?IdPaxina=62714), based on an EPC levels increase >200% in patients with acute ischemic stroke who showed good functional outcome according to previous studies^8^. The minimum sample size calculated to detect this effect was made accepting an alpha level of 5% and an 80% power.

### Statistical tests for univariate analysis

Results were expressed as percentages for categorical variables and as mean (SD) or median and range (25^th^ and 75^th^ percentiles) for continuous variables depending on whether their distribution was normal or not. The Kolmogorov-Smirnov test was used to assess normality. Proportions were compared using the chi-square or Fisher test, while the continuous variables between groups were compared with the Student’s t or the Mann-Whitney tests depending on whether their distribution was normal or not. Bivariate correlations were performed using Pearson’s coefficient (normally distributed variables) or Spearman coefficient (variables without normal distribution).

### Statistical tests for multivariate analysis

The association of EPCs and good functional outcome (mRS ≤ 2 at 12 months) was assessed using logistic regression analysis; the influence on residual ICH volume was assessed by multiple linear regression models. Both logistic regression analysis and multivariable linear regression models were adjusted for those variables with a proven biological relevance for each endpoint in order to avoid spurious associations. Residual plots were examined to detect potential non-linear relationships between the outcome variable and continuous independent variables. Results were expressed as adjusted odds ratios (ORs) or Beta estimate with the corresponding 95% confidence intervals (95% CI).

## Additional Information

**How to cite this article**: Pías-Peleteiro, J. *et al*. Increased Endothelial Progenitor Cell Levels are Associated with Good Outcome in Intracerebral Hemorrhage. *Sci. Rep*. **6**, 28724; doi: 10.1038/srep28724 (2016).

## Figures and Tables

**Table 1:** Baseline clinical characteristics, vascular risk factors, stroke subtype, biochemical parameters and neuroimaging findings in patients with good or poor outcome at 12 months.

	Good Outcome n = 19	Poor Outcome n = 27	p
Age (years)	67.7 ± 10.7	76.1 ± 9.8	0.010
Male, (%)	73.7	70.4	0.806
Time from stroke onset, h	5.1 ± 4.5	4.4 ± 4.8	0.562
Vascular risk factors			
History of hypertension, (%)	63.2	63.0	0.989
History of diabetes, (%)	10.5	11.1	0.950
History of atrial fibrillation, (%)	10.5	25.9	0.195
History of hyperlipidemia, (%)	36.8	28.5	0.363
Smoking habit, (%)	10.5	3.7	0.356
Alcohol consumption, (%)	21.1	14.8	0.583
Biochemistry and vital signs at admission			
Body temperature (°C)	36.4 ± 0.8	36.1 ± 0.5	0.110
Systolic blood pressure (mm Hg)	165.9 ± 29.6	162.2 ± 27.9	0.665
Diastolic blood pressure (mm Hg)	88.2 ± 21.0	86.4 ± 21.2	0.781
Glucose levels (mg/dL)	123.0 ± 34.2	140.2 ± 37.8	0.121
Fibrinogen (mg/dL)	407.7 ± 98.4	451.5 ± 108.0	0.174
Leucocytes (×10^3^/mL)	9.3 ± 2.2	10.2 ± 3.7	0.344
Platelets (×10^3^/mL)	232.9 ± 44.1	232.1 ± 68.1	0.965
INR	1.2 ± 0.5	1.6 ± 1.2	0.077
Neuroimaging findings			
ICH volume at admission (cc)	15.4 ± 12.6	42.3 ± 36.6	<0.0001
Edema volume at admission (cc)	24.7 ± 14.3	51.4 ± 40.5	0.007
Ventricular extension, (%)	15.8	55.6	<0.0001
Leukoaraiosis, (%)	5.3	51.9	<0.0001
Clinical characteristics			
NIHSS at admission	7 [5, 10]	13 [9, 16]	<0.0001
Diagnosis			
Topographic:			0.847
– Deep, (%)	63.2	66.7	
– Lobar, (%)	36.8	33.3	
Etiologic:			0.271
– Hypertensive, (%)	63.1	44.0	
– Amyloid, (%)	31.6	32.0	
– Anticoagulants, (%)	0	16.0	
– Undetermined, (%)	5.3	8.0	
Number of EPCs (CD34^+^/CD133^+^/KDR^+^)			
EPCs at admission	14.2 ± 5.1	10.9 ± 6.1	0.108
EPCs at 72 hours	18.2 ± 5.5	11.2 ± 7.3	0.003
EPCs at day 7	22.7 ± 5.3	13.5 ± 9.1	<0.0001

INR: International Normalized Ratio; ICH: Intracerebral hemorrhage; NIHSS: National Institute of Health Stroke Scale.

**Table 2:** Crude and adjusted OR of good outcome at 12 months for EPCs numbers at 72 hours and day 7.

	OR (CI95%), p	OR (CI95%), adjusted p
EPCs at 72 hours	1.18 (1.04 to 1.34), p = 0.009	1.10 (0.95 to 1.29), p = 0.189
EPCs at day 7	1.31 (1.09 to 1.58), p = 0.002	1.15 (1.01 to 1.35), p = 0.039
NIHSS at admission		0.94 (0.81 to 1.09), p = 0.405
ICH volume at admission		0.98 (0.93 to 1.03), p = 0.383
Age		0.95 (0.86 to 1.04), p = 0.295

NIHSS: National Institute of Health Stroke Scale; ICH: Intracerebral hemorrhage.
